# Hepatocellular Carcinoma Metastasis to the Orbit in a Coinfected HIV+ HBV+ Patient Previously Treated with Orthotopic Liver Transplantation: A Case Report

**DOI:** 10.1155/2011/549270

**Published:** 2011-12-20

**Authors:** S. Guerriero, G. Infante, E. Giancipoli, S. Cocchi, M. G. Fiore, D. Piscitelli, N. Cardascia

**Affiliations:** ^1^Department of Ophthalmology and ORL, Bari University, 70124 Bari, Italy; ^2^U.O. Infectious Disease, P.O. Bisceglie, 70052 Bisceglie, Italy; ^3^Department of Infectious and Tropical Disease, Modena and Reggio Emilia University, 41121 Modena, Italy; ^4^Department of Anatomopathology, Bari University, 70124 Bari, Italy

## Abstract

Hepatocellular carcinoma rarely metastasizes to the orbit. We report a 45-year-old male, HBV+, HIV+, with a past history of a liver transplant for ELSD (end-stage liver disease) with hepatocellular carcinoma and recurrent HCC, who presented with proptosis and diplopia of the left eye. CT scans of the head revealed a large, irregular mass in the left orbit causing superior and lateral destruction of the orbital bone. Biopsy specimens of the orbital tumor showed features of metastatic foci of hepatocellular carcinoma. Only 16 other cases of HCC metastasis to the orbit have been described in literature, and this is the first case in a previously transplanted HIV+, HBV+ patient.

## 1. Introduction

Hepatocellular carcinoma (HCC) accounts for 85–90% of all primary liver cancers. Each year, approximately half a million people worldwide are newly diagnosed with HCC. The majority of HCC cases develop on a substrate of chronic hepatitis B (HBV) and C virus (HCV) infection. 

ESLD (end-stage liver disease) and HCC are increasing causes of mortality in human-immunodeficiency-virus- (HIV-) seropositive patients. Coinfection with HIV may accelerate the progression of chronic HBsAg-positive hepatitis to cirrhosis and may foster the rapid progression of regenerative nodules to dysplastic nodules and HCC. OLT (orthotopic liver transplantation) in HIV patients with ESLD with or without HCC is an emerging option in this set of patients, especially since the administration of HAART can guarantee a good viral-immunological framework.

Approximately 50%–75% of patients with HCC develop metastases during the disease course [1]. The most common sites of metastatic disease are the lung (41.4–51.6%), the regional lymph nodes (26.7–37.9%), and less commonly, the adrenal glands (5.8–10.9%), bones (4.8–8.8%), gallbladder (4.3–11.6%), and peritoneum (4.0–10.9%) [2]. Orbital metastases from HCC are extremely rare, and only 16 cases have been reported in the literature.

We report a case of orbital metastasis from HCC in a 45-year-old HCV+, HIV+ male with a past history of a liver transplant for hepatocellular carcinoma, and recurrent HCC subjected to multiple therapies.

## 2. Case Report

**Figure 1:**
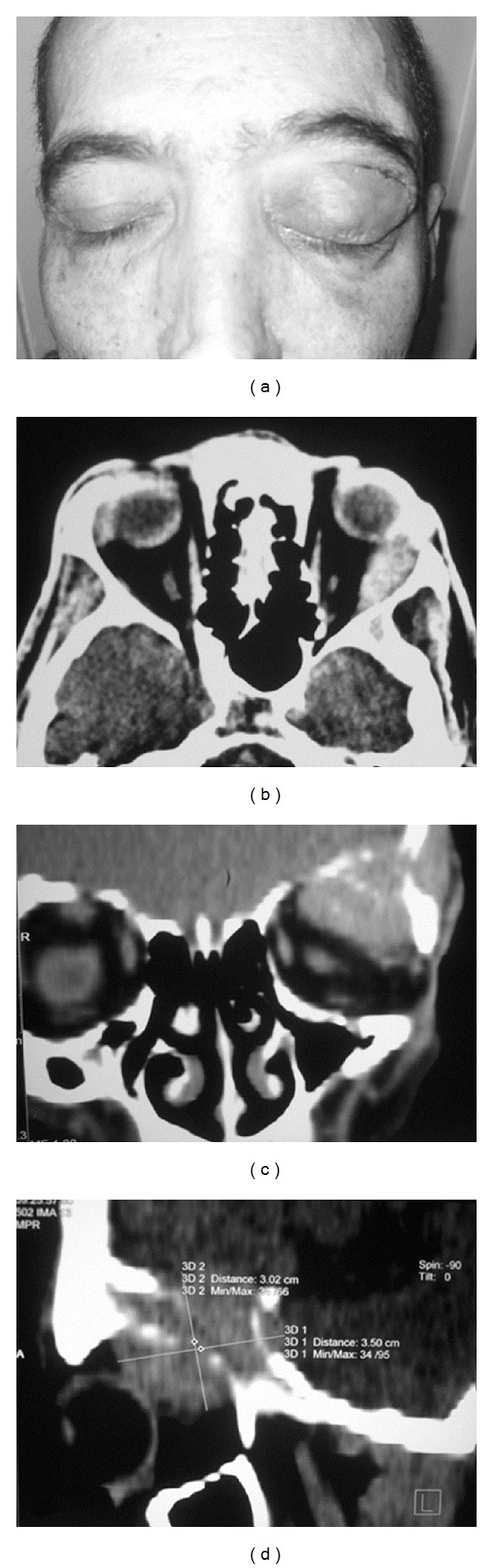
(a) Proptosis of the left eye, (b), (c), and (d) computed tomography (CT) scans of the orbit that showed an extraconal mass involving the superior and lateral wall of the left orbit with destruction of the orbital bone, incorporating the lateral rectus, with calcifications.

A 45-year-old man presented to our department in December 2010 with proptosis of the left eye lasting about one month (Figure 1(a)). He complained of diplopia but no pain or redness. His right eye was unremarkable. His best corrected visual acuity was 20/20 in both eyes. On examination, he had a 6 mm proptosis in the left eye, and an inferior displacement of the eye with superior sulcus fullness. A solid mass was palpable in the superior part of the orbit, not separable from the superior orbital rim. The left eye motility was restricted in the upward and leftward gaze.

 His past medical history was positive for HIV and HBV infection since 1986 and consequent cirrhosis and hepatocellular carcinoma (HCC). In July 2006, he had undergone a liver transplant for ESLD with concomitant HCC, with a marginal organ. Histologic evaluation of the native liver pointed out the presence of a cancer thrombus within the right branch of the portal vein.

 In December 2008, he was subjected to a partial surgical resection of the transplanted liver for a recurrence of HCC. He started therapy with sorafenib in 2009, but took the drug irregularly and reduced the dosage for the concomitant occurrence of diarrhea.

 He presented a new liver and pulmonary recurrence in December 2009. The liver recurrence was treated with selective transarterial chemoembolization, while the pulmonary metastases were treated by local resection of the middle lobe of the left lung. After histological examination of the pulmonary lesions confirmed the HCC recurrence, the patient started systemic chemotherapy with liposomial doxorubicin. In July 2010, the patient presented a new HCC recurrence to the lungs and liver and therefore underwent level II chemotherapy with 5-FU and subsequently with capecitabine. In December 2010, he presented with proptosis and diplopia of the left eye, and AFP was high: 3750 ng/mL. On the suspicion of an orbital metastasis of HCC, the patient underwent computed tomography (CT) scans of the orbit that showed an extraconal mass involving the superior and lateral wall of the left orbit with destruction of the orbital bone, incorporating the lateral rectus, with calcifications (Figures 1(a), 1(c), and 1(d)). Percutaneous incisional biopsy of the lesion was performed.

## 3. Pathological Findings

**Figure 2:**
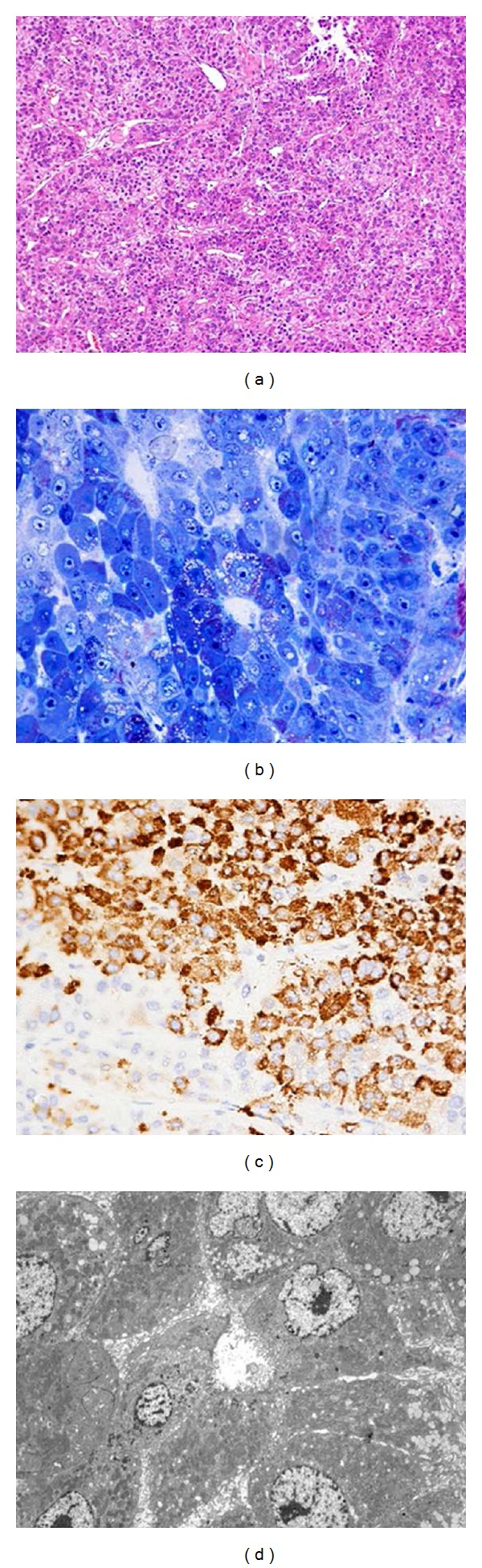
(a) The tumor is composed of cords of cells resembling hepatocytes surrounded by a looser hypocellular fibrous stroma containing prominent sinusoidal vessels. (E.E, 100x). (b) Semithin section: polygonal cells in trabecular pattern, with large nuclei and prominent nucleoli and many intracytoplasmic small vesicles (Polychr. sec. Tolivia, 400x). (c) The neoplastic cells showed strong and diffuse immunoreactivity for OCH1E5. (I.I.C., 400x). (d) Electron microscopic appearance: malignant hepatocytes with numerous mitochondria, abundant glycogen, and large nuclei with prominent nucleoli. Bile canaliculi with stubby microvilli and cell junctions were also evident (TEM, 1800x).

Microscopic examination showed a diffuse infiltrate of large polygonal cells with a finely eosinophilic cytoplasm and roundish-oval hyperchromatic nuclei containing one or a few large nucleoli. The growth pattern was trabecular with the formation of occasional well-defined lumina. The nuclear pleomorphism and mitotic activity were moderate (Figure 2(a)). The neoplastic cells stained positively with periodic acid Schiff (Figure 2(b)) and showed a strong, diffuse immunoreactivity for OCH1E5 and CK20 (Figure 2(c)). CK7, CEA, and *α*-fetoprotein were negative.

At electron microscopy, the neoplastic cells were fairly uniform in shape, size, and structure. They contained numerous mitochondria, a well-developed rough endoplasmic reticulum, intracytoplasmic bile products, and abundant glycogen. Bile canaliculi with stubby microvilli and cell junctions were also evident (Figure 2(d)). The patient was treated with local radiotherapy.

## 4. Discussion

A mass in the orbit is rarely a case of metastasis, featured in only 3–9% of all orbital tumors [3]. Moreover, the most common primary sites of orbital metastases are the breast, the lung, the genitourinary, and the gastrointestinal tract [3]. Clinically diagnosed cases of HCC to the orbit are rare; only 16 cases of HCC metastasizing to the orbit have been reported. The mechanism of metastasis to the orbit is difficult to determine. A hematogenous route, as for other primary neoplasms of the abdomen, may be suspected [4]. Tumor cells may circulate through the vena cava, beyond the pulmonary filter to the heart, and finally being distributed to the orbital region through the arterial systemic circulation [2]. Other authors have reported that tumor cells can reach the head and the neck by bypassing the lungs, possibly through the vertebral venous plexus of Baston [5]. In the present case, the presence of pulmonary metastases leads us to speculate that the first pathway, through the pulmonary filter, may have contributed to this orbital metastasis. Furthermore, when studying the establishment of orbital metastases, we must consider the possibility of infiltration to the orbit secondary to metastasis to the orbital bones [6]. In our patient, there was marked cranial destruction, suggesting the possibility of extension to the orbit secondary to cranial metastases.

The clinical manifestations of metastatic carcinoma are proptosis, ophthalmoplegia, diplopia and acquired strabismus, conjunctival hyperemia, pain, and diminished vision if the tumor infiltrates the choroids [2, 3].

The histopathological diagnosis of HCC is based on the presence of large, polygonal cells, a trabecular pattern, and endothelial cuffing. Differential diagnosis must be made for renal cell carcinoma, that can be present with similar cytological features and architecture, and also shows similar staining for cytokeratins [7]. The two may be differentiated by the use of carcinoembryonic antigen and alfa-fetoprotein [8]. Other diseases to be considered in the differential diagnosis are hepatoid carcinoma of the stomach, lung, and ovary, giant cell carcinoma of the lung, and breast carcinoma, [9] as well as melanoma.

The diagnosis of HCC metastases can be confirmed by the use of immunohistochemistry [10]. HCC metastases can be treated by surgery, radiotherapy, and chemotherapy as in our case, but prognosis is generally poor.

Recently, therapy for advanced HCC was revolutionized by the advent of Sorafenib, a multikinase inhibitor with an antiangiogenic activity targeting the VEGF pathway. Rapamycin, also used as an immunosuppressant in this patient, showed a strongly increased antiproliferative activity targeting the m-Tor pathway in HCC, when paired with Nexavar. Two recent reports have pointed out that discontinuous, low-dose treatment with antiangiogenics might elicit tumor invasiveness and metastasis [11–13].

 Many factors in this patient may have contributed to this unusual presentation of HCC metastasis: the immunosuppression caused by HIV infection, the documented invasion of the vascular bed by HCC on removal of the liver, and the possibility of pulmonary metastasis of HCC (previously documented in a series of 11 patients with rare brain metastases of HCC, in which lung metastasis was present in 91%).

Although orbital metastases are rare, the ophthalmologist should be aware of this clinical form and prognostic implications (especially in patients previously treated with antiangiogenic therapy) and make efforts to palliate the patient's symptoms as far as possible.
